# The Role of Bone Marrow Cells in the Phenotypic Changes Associated with Diabetic Nephropathy

**DOI:** 10.1371/journal.pone.0137245

**Published:** 2015-09-04

**Authors:** Guang Yang, Qingli Cheng, Sheng Liu, Jiahui Zhao

**Affiliations:** Department of Geriatric Nephrology, Chinese People’s Liberation Army General Hospital, State Key Laboratory of Kidney Disease, Beijing, China; Children's Hospital Boston/Harvard Medical School, UNITED STATES

## Abstract

The aim of our study was to investigate the role of bone marrow cells in the phenotypic changes that occur in diabetic nephropathy. Bone marrow cells were obtained from either streptozotocin-induced diabetic or untreated control C3H/He mice and transplanted into control C3H/He mice. Eight weeks after bone marrow cell transplantation, renal morphologic changes and clinical parameters of diabetic nephropathy, including the urine albumin/creatinine ratio and glucose tolerance, were measured *in vivo*. Expression levels of the genes encoding α1 type IV collagen and transforming growth factor-β1 in the kidney were assayed. Our results demonstrated that glucose tolerance was normal in the recipients of bone marrow transplants from both diabetic and control donors. However, compared with recipients of the control bone marrow transplant, the urinary albumin/creatinine ratio, glomerular size, and the mesangial/glomerular area ratio increased 3.3-fold (p < 0.01), 1.23-fold (p < 0.01), and 2.13-fold (p < 0.001), respectively, in the recipients of the diabetic bone marrow transplant. Expression levels of the genes encoding glomerular α1 type IV collagen and transforming growth factor-β1 were also significantly increased (p < 0.01) in the recipients of the diabetic bone marrow transplant. Our data suggest that bone marrow cells from the STZ-induced diabetic mice can confer a diabetic phenotype to recipient control mice without the presence of hyperglycemia.

## Introduction

The prevalence of chronic kidney disease in China is 10.8%. Diabetes is one of the risk factors independently associated with chronic kidney disease [1]. About 25% to 40% of patients with diabetes will develop diabetic nephropathy (DN), characterized by histological changes or a progressive decline in the glomerular filtration rate, within 20 to 25 years of the onset of type 1 or type 2 diabetes mellitus. ECM accumulation and mesangial cell proliferation are pathologic hallmarks or phenotypic changes associated with DN [2].

Hyperglycemia has been implicated in the development of the phenotypic changes associated with DN [3]. Several mechanisms contribute to these phenotypic changes, including high mitochondrial oxidative stress, metabolic pathways, chronic inflammation, immune-related podocyte injury and participation of cytokines [3–5]. However, the Diabetes Control and Complications Trial observed that some patients are resistant to DN development despite years of poor blood glucose control, whereas 26% of the patients still progressed to DN despite adequate blood glucose control. These observations indicated that other factors, in addition to hyperglycemia, contribute to the progression of DN [6].

Mesenchymal stem cells, also known as multipotent mesenchymal stromal cells, are self-renewing cells that can be found in almost all postnatal organs and tissues. Mesenchymal stem cells are most frequently isolated from bone marrow (BM) and are also known as BM stem cells. It has been previously demonstrated that BM stem cells contribute to renal regeneration in animal models of DN. Moreover, the function of BM stem cells may be affected by hyperglycemia associated with diabetes [7]. A previous study demonstrated that naive syngeneic B6 mice developed albuminuria and severe glomerular lesions in the absence of hyperglycemia when transplanted with BM cells from db/db mice with established DN [8]. However, the db/db mouse is a spontaneous diabetic model; thus, it is difficult to identify whether the DN phenotypic changes were induced by genetic factors or were acquired characteristics of BM cells. To elucidate the role of BM cells in the phenotypic changes associated with diabetic nephropathy, the genetic components of diabetes should be excluded from the animal model. To determine whether C3H/He mice were vulnerable to hyperglycemia, the effects of high ambient glucose concentrations on mesangial cells isolated from C3H/He and C57BL/6 mice were compared. In addition, we established a streptozotocin (STZ)-induced diabetic model using C3H/He mice. Finally, the role of BM cells in diabetic phenotypic changes was investigated in BMT recipient C3H/He mice.

## Materials and Methods

### Animals

C3H/He and C57BL/6 female mice (six to eight weeks of age) were purchased from the experimental animal research unit of Beijing Union Medical College, China Academy of Medical Sciences. Mice were maintained in a temperature-controlled (25°C) facility with a strict 12 h light/dark cycle and were given *ad libitum* access to food and water. This project was approved by the Animal Care and Use Committee of the Chinese People’s Liberation Army General Hospital (2013-XC-12). This study was carried out in strict accordance with the recommendations in the Guide for the Care and Use of Laboratory Animals of the National Institutes of Health. Mice were euthanized with CO_2_ under isoflurane anesthesia and all efforts were made to alleviate suffering.

### Isolation and Cloning of Glomerular Mesangial Cells

The kidneys of control C3H/He and C57BL/6 mice were perfused with phosphate-buffered saline (PBS) solution. The renal cortex was removed and incubated in 0.1% collagenase/PBS buffer for glomeruli isolation. Glomeruli were separated from tubules and arterioles under a microscope. Microdissected glomeruli were placed into wells of fibronectin-coated tissue culture plates and cultured in dulbecco’s modified eagle’s medium and Ham’s F-12 nutrient mixture medium (3:1; Gibco BRL, Invitrogen Corporation, USA) supplemented with 20% fetal bovine serum (Gibco BRL), 1.0mM glutamine (Gibco BRL), 0.075% Na_2_HCO_3_ (Gibco BRL), 100 μg/ml penicillin-streptomycin (100 units/ml; Gibco BRL), and trace elements (Biosource, USA). The culture media glucose concentration was 5.5 mM. Glomerular mesangial cells were characterized as previously described [9]. Individual clusters of outgrowing cells were isolated with cloning rings and trypsinized, followed by single cell cloning and propagation.

### Cell Experimental Design

To investigate the effects of high ambient glucose on mesangial cells from C3H/He and C57BL/6 mice, cells were examined following treatment with 5.5 mM or 30 mM glucose in dulbecco’s modified eagle’s medium and Ham’s F-12 nutrient mixture medium (3:1; 10% fecal calf serum; Table 1). Cell culture medium was supplement with ascorbic acid (50μg/ml, Sigma, USA) and beta-aminopropionitrile (80μg/ml, Sigma).

**Table 1:** Collagen Type I and IV assay in mesangial cell culture supernatants.

Mesangial cells	Collagen Type IV (μg/ml/10,000 cells)	Collagen Type I (μg/ml/10,000 cells)
C57 Mes. + 5.5 mM Glucose	0.0114±0.0013	0.0237±0.0095
C57 Mes. + 30 mM Glucose	0.0123±0.0018*	0.0243±0.0084
C3H Mes. + 5.5 mM Glucose	0.0562±0.0011	0.0189±0.0116
C3H Mes. + 30 mM Glucose	0.0953±0.0019** #	0.0321±0.0089

*p < 0.05, compared with C57 mesangial cells in 5.5 mM glucose

**p < 0.01, compared with C3H mesangial cells in 5.5 mM glucose; and

#p < 0.01, compared with C57 mesangial cells in 30 mM glucose.

### Collagen Types I and IV Assay

Mesangial cell numbers were determined, and the cell supernatants were collected on the 14th day after treatment with different glucose concentrations. Collagen type I and IV ELISAs were performed. Briefly, standards were plated in the linear range of the curve, ranging from 0.023 ng/μl to 1.5 ng/μl for collagen type I (Collaborative Biomedical Products, USA) and from 0.023 ng/μl to 3 ng/μl for collagen type IV (Collaborative Biomedical Products). To measure collagen type I levels, the supernatants were added to 96-well-plates, which were incubated at 37°C for 2 h, followed by 30 min at room temperature (27°C) in blocking solution (0.05% Tween-20, 0.25% bovine serum albumin in PBS buffer) and an overnight incubation at 4°C with a rabbit anti-mouse type I collagen polyclonal antibody (1:2,000; Biodesign Int, USA). Samples were subsequently incubated with a biotinylated goat anti-rabbit IgG polyclonal antibody (1:2,000; Biosource International, USA) for 2 h at room temperature. For collagen type IV, the samples were similarly processed, but a rabbit anti-mouse collagen type IV polyclonal antibody (1:3,000; Biodesign Int.) was used. Final values were expressed as nanograms per 10,000 cells for collagen types I and IV. Mesangial cell mRNA was extracted with Tri-Reagent (Sigma, USA). Reverse transcription (RT)-PCR was performed and *Gapdh* was used as a housekeeping gene. The primer sequences for *Col4a1*, the gene encoding α1 type IV collagen (484 bp), were as follows: forward: 5’-CACCATAGAGAGAAGCGAGATGTTC-3’; reverse, 5’-GGCTGACGTGTGTTCGC-3’.

### Experimental and control groups

Four experimental groups were divided as follows: Control C3H/He mice, C3H/He mice with diabetes, BM transplant (BMT) with BM from control C3H/He mice and BMT with BM from diabetic C3H/He mice. There were 5 mice in each group. Animals are grouped by random number table method.

### Induction of Diabetes in Mice

Diabetes was induced in six-week-old female C3H/He mice by intraperitoneal (IP) injections of STZ (Sigma). Briefly, STZ was dissolved in an ice-cold citrate buffer (pH 4.6) and injected IP (100 mg/kg body weight/day) twice per week until hyperglycemia (> 16.7mM) was achieved and maintained for at least one week. The control mice received an IP injection of the citrate buffer without STZ.

### BM Cell Transplantation

BM was obtained from diabetic and control C3H/He mice. Femurs and tibiae were collected and the BM cells were harvested and resuspended in sterile PBS buffer. Control C3H/He mice were used as BM transplant (BMT) recipients. After lethal irradiation (950 cGy), the mice were anesthetized using a ketamine, xylaxine, and acepromazine mixture. The mice were then divided into two groups and injected intravenously with 5 × 10^6^ BM cells from diabetic or control mice. There were no changes in the general health status of the recipient mice before they were sacrificed for the studies.

### IP Glucose Tolerance Test

IP glucose tolerance tests were performed as previously described [10]. Briefly, the mice were fasted overnight (12 h) and were then injected intraperitoneally with glucose (2.0 g/kg body weight with 25% glucose in a sterile solution). Blood samples from the mice were collected for glucose quantification at 0, 15, 30, 60, and 120 min after injection.

### Urine Albumin/Creatinine Ratio

Urine from the mice was collected into siliconized tubes. For the albumin assay, the urine was diluted 1:1,000 and measured in duplicate with the Mouse Albumin ELISA Quantitation Kit (Bethyl Laboratories Inc., USA). Optical density values were obtained using the MRX microplate reader (Dynex Technologies Inc., USA) at a wavelength of 450 nm. Albumin values (ng/ml) were determined as the mean of duplicate values. The urine creatinine (mg/dl) was measured using the alkaline picrate (Jaffé) method. Creatinine LiquiColor Test was purchase from StanBio Lab Inc. (USA). Urine creatinine assay was performed on spectrophotometer (Bio-Rad, capable of absorbance readings at 510 nm) in accordance with the manual steps. The urinary albumin/creatinine ratio (mg/g) was calculated for each animal.

### Renal Tissue Preparation

The recipient mice were sacrificed eight weeks after BMT. After perfusion with PBS buffer, the upper and lower poles of the left kidneys were removed. The glomeruli were isolated under a microscope for subsequent real time RT-PCR analysis. The remaining kidney was cut into small pieces and either prepared for frozen sectioning or fixed in 4% formalin, embedded in methacrylate, and sectioned for pathological assessment.

### Renal Pathologic Study

Sections of renal cortical tissue (4 μm) were stained using the Periodic Acid–Schiff method. Digital images of 50 consecutive, randomly chosen glomeruli from each mouse were recorded with an Olympus BH-2 microscope and a MicroImage A209 RGB color video camera. Total glomerular area (μm^2^) and mesangial area (μm^2^) were obtained using the MetaMorph image analysis computer program (Universal Imaging Corporation, USA).

Collagen type IV expression was evaluated using immunofluorescence microscopy. Briefly, renal frozen tissues were cut into 4-μm sections and mounted on silane-coated glass slides. The slides were washed and incubated at room temperature in PBS containing 3% bovine serum albumin, 10% normal goat serum, and purified rabbit anti-mouse type IV collagen IgG (1:160; Biodesign, USA). After one hour, the slides were washed, incubated for one hour at room temperature with FITC-labeled goat anti-rabbit IgG (Zymed, USA), and washed again, and cover slips were applied. The negative control consisted of adjacent sections exposed only to the secondary antibody.

### Analysis of the Expression Levels of the Genes Encoding TGF-β1 and Collagen Type IV mRNA by Real Time RT-PCR Assay

Glomeruli were isolated from the BMT recipients. Equal amounts of RNA isolated from each sample were reverse transcribed and amplified with primers specific to *Col4a1* and *Tgfb1*, the genes encoding α1 type IV collagen and TGF-β1, respectively, using the TaqMan one step real time PCR master mix reagents kit and the ABI Prism 7700 sequence detection system (Perkin Elmer Applied Biosystems, USA). A standard curve using the 18S rRNA amplicon was developed. 18S rRNA levels and *Col4a1* and *Tgfb1* mRNA levels were expressed as the number of copies per microgram of total RNA. The primers were as follows: for *Col4a1*, forward: 5'-CACCATAGAGAGAAGCGAGATGTTC, reverse: 5'-GGCTGACGTGTGTTCGC; and for *Tgfb1*, forward: 5'-ACTGGAGTTGTACGGCAGTGG, reverse: 5'-GCAGTGAGCGCTGAATCGA. 4,7,2’-trichloro-7’-phenyl-6-carboxyfluorescein-labeled 5'-AAGCCCACGCCATCCACCTTGA was used as the probe for *Col4a1* amplification. 6-carboxyfluorescein-labeled 5'-TGAACCAAGGAGACGGAATACAGGGCT was used as the probe for *Tgfb1* amplification. The TaqMan Ribosomal RNA Control Reagents kit was used to measure the expression of 18S rRNA. The *Col4a1* and *Tgfb1* mRNA expression levels were normalized to 18S rRNA levels in recipient mouse samples.

### Statistical Analysis

Data are expressed as mean ± standard deviation. An unpaired Student’s *t* test was used to compare the means of two groups. Statistical significance was defined as p < 0.05.

## Results

### Collagen Type I and IV Assay in Mesangial Cell Culture Supernatants

The levels of collagens type I and IV secreted by glomerular mesangial cells from the different mouse models under different glucose conditions were measured. Treatment with 30 mM glucose resulted in a 1.2-fold and 1.7-fold increase in collagen type IV secretion from mesangial cells isolated from C57BL/6J and C3H/He mice, respectively, compared with that of the respective 5.5 mM glucose treatment groups (Table 1). Under these conditions, the secretion of collagen type IV from mesangial cells isolated from C3H/He mice was higher than that from the C57BL/6J mesangial cells (p < 0.01). However, there were no differences in collagen type I secretion between groups.

### An Analysis of Expression Levels of the Gene Encoding Collagen Type IV in Mesangial Cells

**Fig 1:**
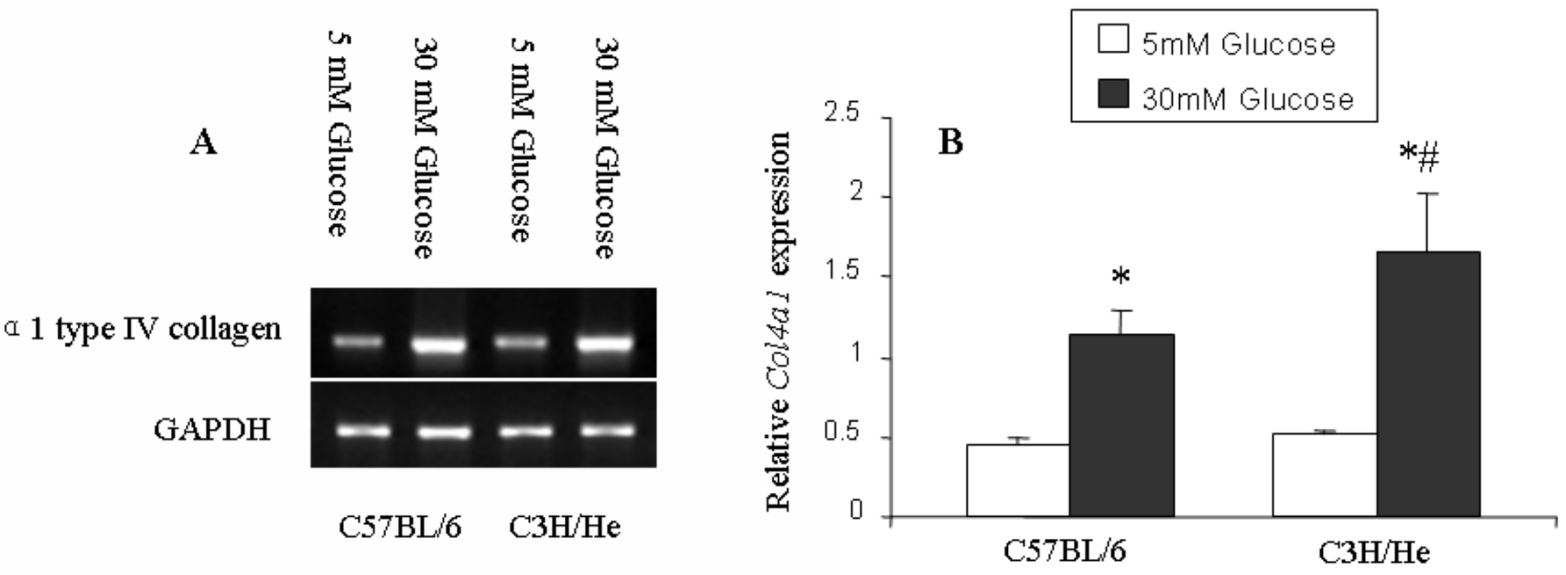
Changes in *Col4a1* mRNA expression in mesangial cells. **(**A) mRNA expression of *Col4a1*. Hyperglycemia induces *Col4a1* expression in mesangial cells from both C3H/He and C57BL/6 mice. (B) *Col4a1* mRNA expression (expressed as a ratio of *Col4a1* to *GAPDH* expression) was increased following treatment with 30 mM glucose (closed bars), compared with that in 5.5 mM glucose (open bars), in both strains (*p < 0.05, n = 3). *Col4a1* expression at 30 mM glucose was significantly higher in C3H/He mesangial cells compared with that in C57BL/6 mesangial cells ^(#^p < 0.01, n = 3).

There was no difference in expression levels of the gene encoding collagen type IV, *Col4a1*, between C3H/He and C57BL/6 mesangial cells at 5.5 mM glucose. However, *Col4a1* expression increased in both strains when the glucose concentration in the culture medium increased from 5.5 mM to 30 mM and was markedly higher in C3H/He mesangial cells (3.2-fold higher in C3H/He mesangial cells versus 2.6-fold higher in C57BL/6 mesangial cells; p < 0.01; Fig 1).

### Analysis of Glycemia and Albuminuria Levels

**Fig 2:**
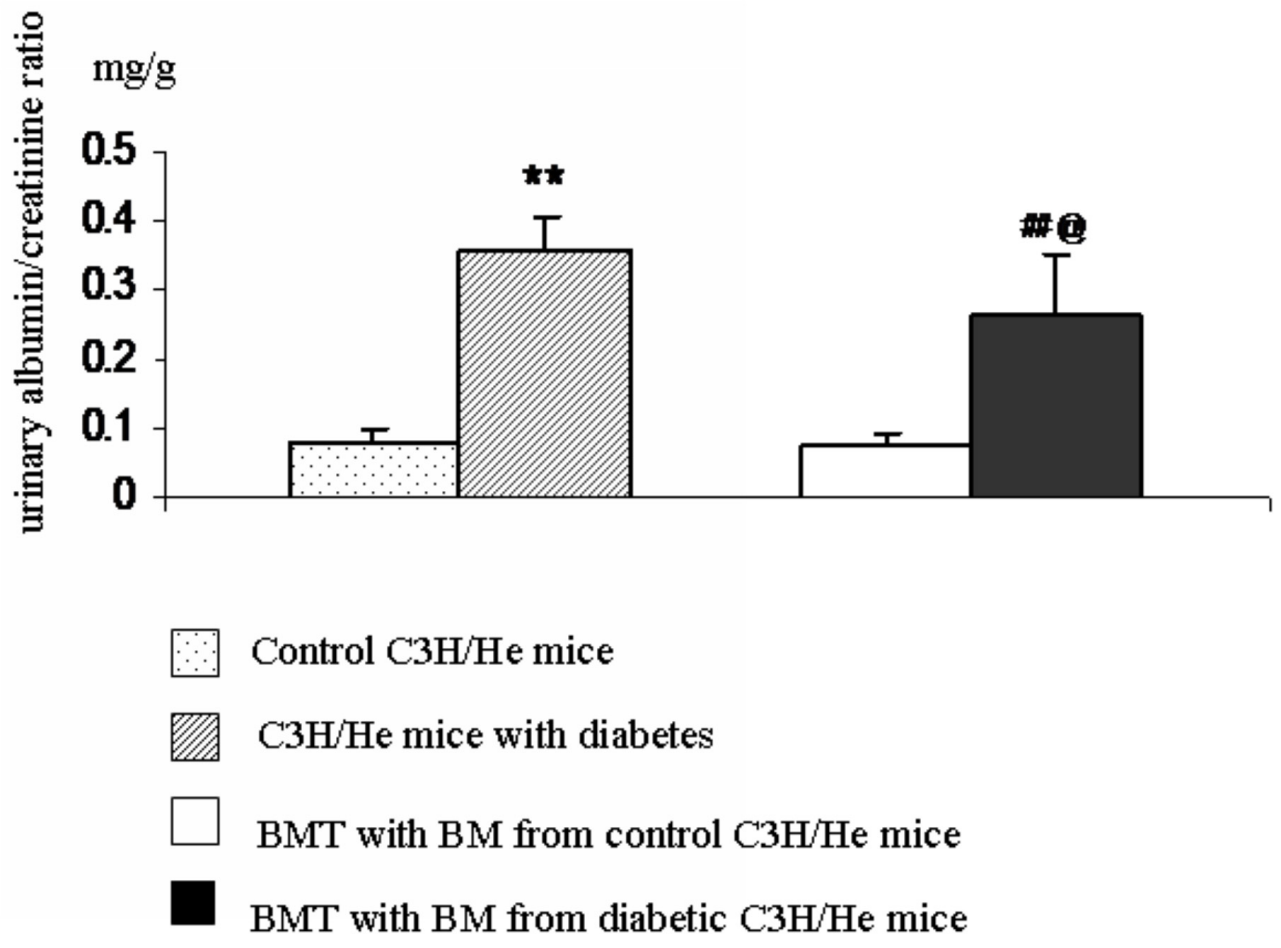
Analysis of albuminuria levels. The urinary albumin/creatinine ratio in the diabetic recipients was 3.3-fold higher than that of the control recipients (^##^p < 0.01, n = 5), while they were lower than diabetic C3H/He mice (^@^p < 0.05, n = 5). BMT, bone marrow transplant; BM, bone marrow.

Mice that received a BMT from either diabetic donors (diabetic recipients) or control donors (control recipients) displayed normal glucose tolerance. Glycemic levels returned to baseline in both groups of mice two hours after the glucose challenge (2.0 g/kg). However, the urinary albumin/creatinine ratio in the diabetic recipients was 3.3-fold higher than that of the control recipients (p < 0.01; Fig 2). While, the urinary albumin/creatinine ratio in the diabetic recipients were still lower than diabetic C3H/He mice (p < 0.05; Fig 2).

### Renal Pathology and Immunopathology

**Fig 3:**
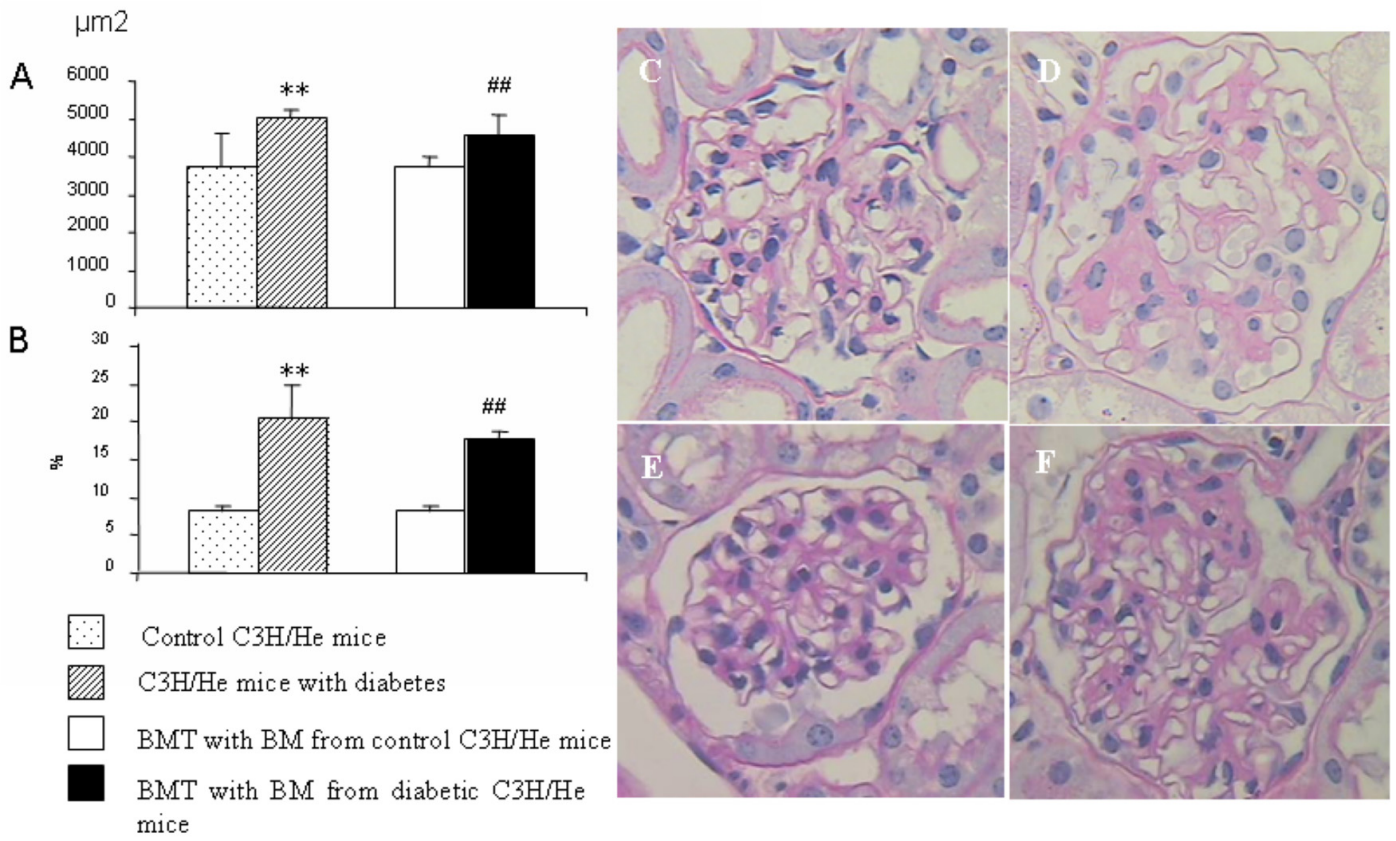
Renal pathology. **(A)** The glomerular area of the diabetic recipients was markedly increased (1.23-fold increase in size; p < 0.01; n = 5). **(B)** The ratio of mesangial to glomerular area also increased in the diabetic recipients (2.13-fold increase, p < 0.001, n = 5). **(C)** The glomeruli of the control C3H/He mice appear normal in morphology and size. **(D)** The glomeruli of C3H/He mice with STZ-induced diabetes are large and have a marked increase in mesangial matrix. **(E)** The glomeruli of the control recipients appear normal in morphology and size. **(F)** The glomeruli of the diabetic recipients are large and have a marked increase in mesangial matrix (Periodic Acid–Schiff stain; original magnification: 400×). BMT, bone marrow transplant; BM, bone marrow.

**Fig 4:**
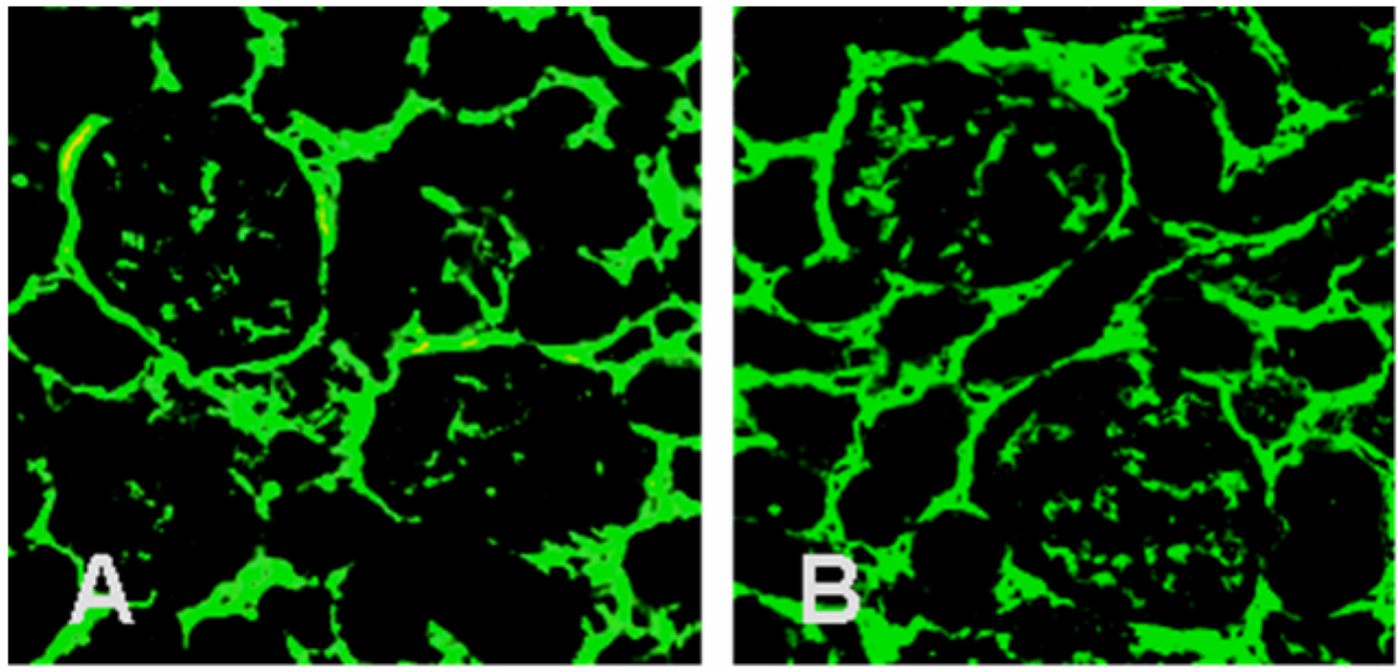
Renal immunopathology. There was a large accumulation of collagen type IV in the glomerular mesangial space of the diabetic recipients. (A) Control recipients. (B) Diabetic recipients (original magnification 400×).

The diabetic recipients developed marked glomerular hypertrophy (1.23-fold increase in size; p < 0.01; Fig 3). The ratio of mesangial to glomerular area also increased 2.13-fold in the diabetic recipients (p < 0.001; Fig 3). Additionally, there was substantial accumulation of collagen type IV in the enlargement of the mesangial spaces, which was detected by immunofluorescence microscopy (Fig 4).

### *Col* *4a* *1* and *Tgfb1* mRNA Levels in Glomeruli

**Fig 5:**
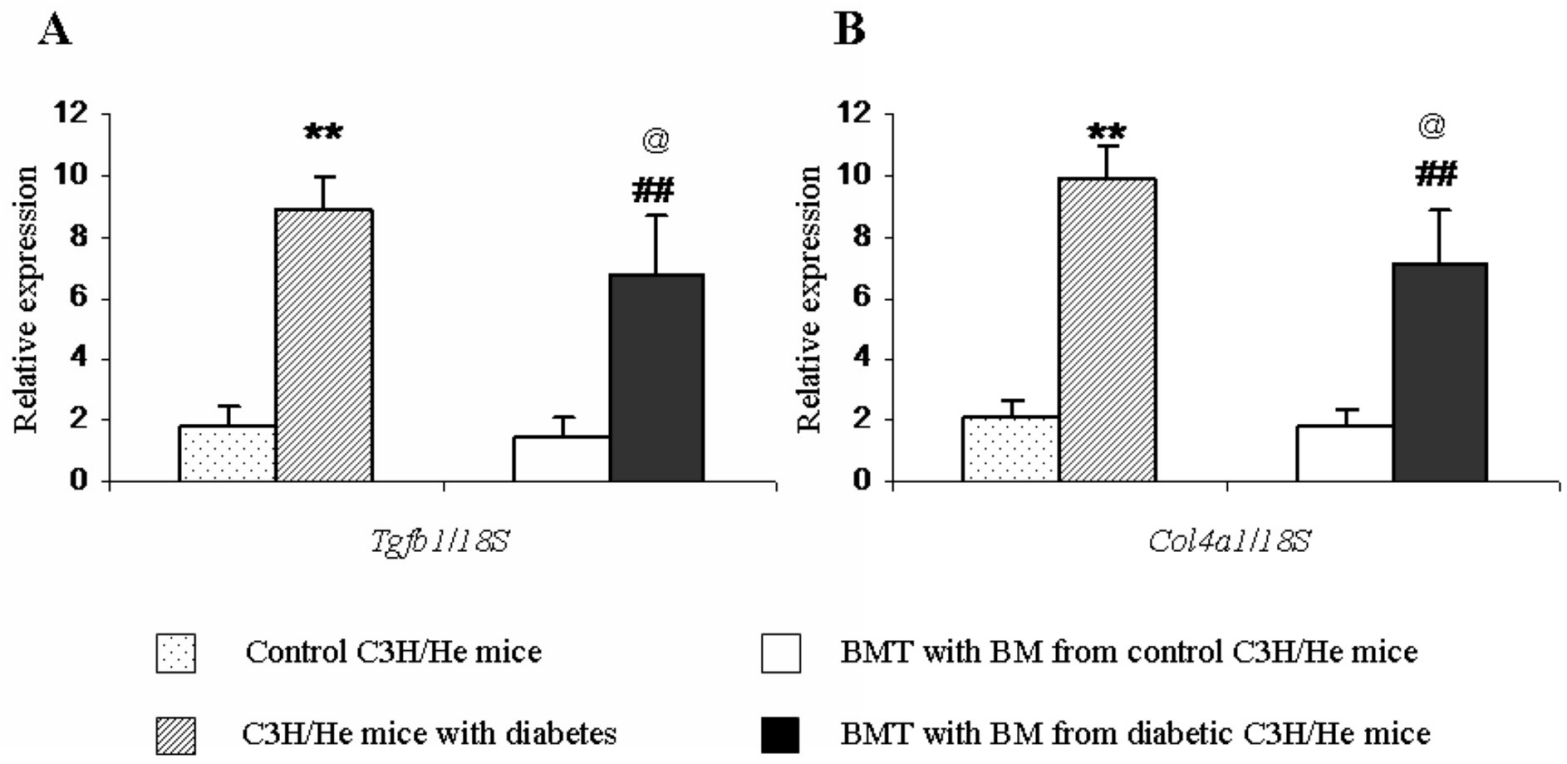
*Col4a1* and *Tgfb1* mRNA levels in the glomeruli. *Col4a1* and *Tgfb1* expression levels were up-regulated in the glomeruli of the diabetic recipients compared with those of the control recipients (^##^p < 0.01; n = 5). While, compared with diabetic C3H/He mice, the *Col4a1 and Tgfb1*mRNA levels were all lower in the diabetic recipients.(^@^p < 0.05; n = 5). BMT, bone marrow transplant; BM, bone marrow.

*Col4a1* and *Tgfb1* expression levels in the glomeruli were measured by real-time RT-PCR. Compared with those of control recipients, there was a significant increase in *Col4a1* mRNA levels in the diabetic recipients (p < 0.01; n = 5). While, compared with diabetic C3H/He mice, the *Col4a1* mRNA levels were lower in the diabetic recipients. (p < 0.05; n = 5; Fig 5). Similarly, glomerular *Tgfb1* expression was also up-regulated in the diabetic recipients (p < 0.01; n = 5). While, compared with diabetic C3H/He mice, the *Tgfb1*mRNA levels were lower in the diabetic recipients. (p < 0.05; n = 5; Fig 5).

## Discussion

ECM accumulation and mesangial cell proliferation are hallmarks in the formation of glomerular and interstitial lesions in DN [2–4]. Hyperglycemia and hemodynamic factors are considered to be major risk factors for the above DN phenotypic changes. In this study, the effects of high ambient glucose concentrations on mesangial cells from C3H/He and C57BL/6 mice were investigated. The results demonstrated that collagen type IV secretion and *Col4a1* mRNA expression increased under hyperglycemic conditions in both strains; however, more dramatic changes were observed in C3H/He mesangial cells. The results indicated that C3H/He mice were more vulnerable to hyperglycemia-induced DN phenotypic changes. After transfer of the BM cells from the STZ-induced diabetic C3H/He mice to the control C3H/He mice, we observed marked glomerular hypertrophy development in the recipient mice accompanied by normal glucose tolerance and the absence of hyperglycemia. Moreover, diabetic recipients displayed albuminuria, increased mesangial ECM, and the accumulation of collagen type IV. These results suggested that BM cells from the STZ-induced diabetic mice could transfer the DN phenotypic changes (such as ECM accumulation in glomeruli) to the control recipients. These results are consistent with a previous study that demonstrated that when BM stem cells from db/db diabetic mice were transplanted into control mice, the recipient mice displayed DN lesions without concomitant changes in blood glucose levels [8]. In addition, Cornacchia et al. observed that a glomerulosclerosis phenotype could be transmitted through BMT from *Os*/+ mouse donors (a model of glomerulosclerosis without diabetes) into congenic ROP +/+ mice (normal glomeruli) [11]. However, as both studies utilized genetic models, we could not distinguish whether the observed changes were due to hereditary factors or to acquired characteristics of BM cells.

How BM cells from STZ-induced diabetic donors transfer DN phenotypic changes to control mice can be clarified based on the following aspects: First, diabetes may affect the functions and biological characteristics of BM cells. Stolzing and colleagues isolated BM stem cells from rats with type 1 diabetes mellitus and studied their proliferative capacity *ex vivo*. They reported that BM stem cell colony size and number were significantly reduced in diabetic rats [12]. In addition, another study found that if BM stem cells were cultured under hyperglycemic conditions, negative effects on colony formation and differentiation were observed [13]. When treated with glyceraldehydes and glycolaldehydes, BM stem cells displayed reduced cell proliferation, increased cell apoptosis, and impaired differentiation into adipogenic, chondrogenic, and osteogenic clones [14]. Additionally, high glucose concentrations reduced the osteogenic and chondrogenic potential of BM stem cells [15]. Similarly, our group previously observed that proliferation capacity, anti-hypoxic properties and the restoration of kidney-derived stem cells were inhibited under hyperglycemic conditions [16]. The mechanisms involved in the toxic effects of hyperglycemia on BM stem cells appear to be related to an imbalance between nitric oxide levels, reactive oxygen species [17], and advanced glycation end-products [12,14].

Moreover, BM cells can affect many aspects of diabetic kidney disease [18–20]. When researchers transplanted BM cells into diabetic mice, 11% of the BM cells engrafted into the damaged kidneys, where they differentiated into endothelial cells and possibly mesangial cells. Other pathological changes included decreases in mesangial thickening, ECM deposition, and macrophage infiltration [20]. BM cells may exert therapeutic effects by mobilizing and migrating to the target organs and subsequently differentiating into the target cell type or through paracrine signaling [7,21]. Moreover, BM cells can interact with the immune system (dendritic cells, T cells, and NK cells) to produce soluble factors and modulate cytokine levels [22]. BM cells can also inhibit the proliferation of B cells and decrease the secretion of IgG via the production of soluble factors [23]. Research from Ben Nasr M et al. further confirms the important role of stem cells in the modulation of inflammatory/immune response. In this research, MSCs could delay allograft rejection in vivo when co-transplanted with islets through multiple immuno-regulatory properties [24].

Thus, we postulated that the function of BM cells from diabetic donor mice was affected by the diabetic environment. Therefore, when we transplanted these dysfunctional BM cells into healthy control mice, the DN phenotype was transferred through direct differentiation, paracrine signaling, or immunomodulatory mechanisms. From our research, the results also indirectly manifested that hyperglycemia is not the only impact factor of diabetic nephrology, even if the blood glucose control in the normal range, also could not completely prevent the occurrence and development of diabetic nephrology. For example, results from ADVANCE research showed that, achieve a glycated hemoglobin value of 6.5% or less, could only get a reduction in the incidence of nephropathy into 4.1% [25]. Another research also show that there were only a little difference in the incidence of microalbuminuria between patients with poor glycemic control and good glycemic control (55% and 54% respectively) [26]. Renal lesions from diabetes could be attributed partly to the direct effects of glycemia, maybe renal lesions in the transplanted mice would be even more aggravated in the presence of hyperglycemia, but this is just our speculation. The exact mechanism requires further investigation.

## Conclusions

Transplantation of BM cells from STZ-induced diabetic mice led to albuminuria, marked glomerular hypertrophy, increased mesangial ECM, and accumulation of collagen type IV without concomitant hyperglycemia in healthy control recipients. These results suggest that BM cells play a key role in the phenotypic changes of DN in diabetic recipients. However, the exact mechanism underlying the transfer of the diabetic phenotype still warrants further research.

## Supporting Information

S1 Table The ARRIVE Guidelines Checklist. (PDF)

S2 Table Raw data of measurements shown in “Fig 1B. Changes in *Col* *4a* *1* mRNA expression in mesangial cells”.
(PDF)

S3 Table Raw data of measurements shown in “Fig 2. Analysis of albuminuria levels”.
(PDF)

S4 Table Raw data of measurements(glomerular and mesangial area) shown in “Fig 3. Renal pathology”.
(PDF)

S5 Table Raw data of measurements shown in “Fig 5. *Col* *4a* *1* and *Tgfb1* mRNA levels in the glomeruli”.
(PDF)
