# Supplementary material for: The Role of Bone Marrow Cells in the Phenotypic Changes Associated with Diabetic Nephropathy
Source: PLoS One. 2015 Sep 4;10(9):e0137245. doi: 10.1371/journal.pone.0137245 (PMC4560440; doi:10.1371/journal.pone.0137245)
Supplement: S2 Table — Changes in Col 4a 1 mRNA expression in mesangial cells”. (PDF) [file pone.0137245.s002.pdf]

Table S2

C57BL/6		C3H/He	
**5mM Glucose**	**30 mM Glucose**	**5mM Glucose**	**30mM Glucose**
0.5163	0.9636	0.5396	1.2028
0.3690	1.3364	0.5041	2.1105
0.4427	1.1500	0.5219	1.6566
