# Supplementary material for: The Role of Bone Marrow Cells in the Phenotypic Changes Associated with Diabetic Nephropathy
Source: PLoS One. 2015 Sep 4;10(9):e0137245. doi: 10.1371/journal.pone.0137245 (PMC4560440; doi:10.1371/journal.pone.0137245)
Supplement: S3 Table — Analysis of albuminuria levels”. (PDF) [file pone.0137245.s003.pdf]

Table S3

Control C3H/He mice mice	C3H/He mice with diabetes	BMT with BM from control C3H/He mice	BMT with BM from diabetic C3H/He mice
0.0696	0.3502	0.0916	0.1052
0.1089	0.3342	0.0783	0.3508
0.0891	0.4124	0.0955	0.3005
0.0810	0.4021	0.0727	0.2494
0.0875	0.2913	0.0705	0.2824
0.0533	0.3772	0.0545	0.3139
