# Supplementary material for: The Role of Bone Marrow Cells in the Phenotypic Changes Associated with Diabetic Nephropathy
Source: PLoS One. 2015 Sep 4;10(9):e0137245. doi: 10.1371/journal.pone.0137245 (PMC4560440; doi:10.1371/journal.pone.0137245)
Supplement: S4 Table — Renal pathology”. (PDF) [file pone.0137245.s004.pdf]

Table S4

Normal C3H/He mice		C3H/He mice with diatetes		BMT with BM from normal C3H/He mice		BMT with BM from diabetic C3H/He mice	
glomerular area(μm^2)	mesangial area(μm^2)	glomerular area(μm^2)	mesangial area(μm^2)	glomerular area(μm^2)	mesangial area(μm^2)	glomerular area(μm^2)	mesangial area(μm^2)
3950.69	364.85	4961.11	759.49	3532.09	330.90	4410.04	863.65
5316.50	494.34	4867.08	947.68	4034.90	331.20	4356.56	684.38
3354.96	245.22	5357.05	1210.27	3334.90	271.89	5242.00	932.12
3056.74	241.46	5219.46	834.17	3924.00	321.62	4959.60	890.58
3031.58	218.09	4734.86	1337.16	3732.69	282.50	3791.01	650.10
