# Supplementary material for: The Role of Bone Marrow Cells in the Phenotypic Changes Associated with Diabetic Nephropathy
Source: PLoS One. 2015 Sep 4;10(9):e0137245. doi: 10.1371/journal.pone.0137245 (PMC4560440; doi:10.1371/journal.pone.0137245)
Supplement: S5 Table — Col 4a 1 and Tgfb1 mRNA levels in the glomeruli”. (PDF) [file pone.0137245.s005.pdf]

Table S4

Normal C3H/He mice		C3H/He mice with diatetes		BMT with BM from normal C3H/He mice		BMT with BM from diabetic C3H/He mice	
Tgfb1/*18S*	*Col4a1*/*18S*	*Tgfb1*/*18S*	*Col4a1*/*18S*	*Tgfb1*/*18S*	*Col4a1*/*18S*	*Tgfb1*/*18S*	*Col4a1*/*18S*
0.553	1.997	8.272	10.400	0.519	1.257	6.833	7.140
1.884	1.573	9.157	8.683	2.130	2.589	4.988	5.354
1.987	1.447	8.647	9.870	1.753	1.917	4.783	5.915
2.465	2.832	10.586	11.288	1.444	1.880	9.363	7.347
1.967	2.746	7.985	9.323	1.894	1.144	7.917	9.935
